# Gastrointestinal complaints in patients with anorexia nervosa in the timecourse of inpatient treatment

**DOI:** 10.3389/fpsyt.2022.962837

**Published:** 2022-08-18

**Authors:** Caroline Riedlinger, Nazar Mazurak, Norbert Schäffeler, Andreas Stengel, Katrin Elisabeth Giel, Stephan Zipfel, Paul Enck, Isabelle Mack

**Affiliations:** ^1^Department of Psychosomatic Medicine and Psychotherapy, University Hospital Tübingen, Tübingen, Germany; ^2^Centre of Excellence for Eating Disorders (KOMET), Tübingen, Germany; ^3^Charité Center for Internal Medicine and Dermatology, Department for Psychosomatic Medicine, Charité-Universitätsmedizin Berlin, Corporate Member of Freie Universität Berlin, Humboldt-Universität zu Berlin, Berlin Institute of Health, Berlin, Germany

**Keywords:** abdominal pain, anorexia nervosa, constipation, eating disorders, gastrointestinal complaints, indigestion

## Abstract

**Background:**

In patients with anorexia nervosa (AN), gastrointestinal (GI) symptoms are common and usually improve during or after nutritional rehabilitation. It is unclear when exactly GI symptoms change in the timecourse of treatment and to which extent. In this study, we analyzed the timecourse of GI symptoms and their relation to disease-specific, demographic, anthropometric, and psychological factors in inpatients with AN.

**Methods:**

In weekly intervals, the Gastrointestinal Symptom Rating Scale (GSRS) was completed, and body weight was measured over a mean of 9.5 weeks in inpatients with AN. A total of four self-report questionnaires assessing psychological factors were completed before and after inpatient treatment. Data from 38 inpatients with AN were analyzed using mixed linear models.

**Results:**

Abdominal pain and constipation improved significantly in the timecourse with 0.085 (*p* = 0.002) and 0.101 (*p* = 0.004) points per week on the GSRS and were predicted to normalize after 13 (*p* = 0.002) and 17 (*p* = 0.004) weeks, respectively. Total GI symptoms tended to normalize after 25 weeks (*p* = 0.079). Indigestion (borborygmus, abdominal distension, eructation, flatulence) was the most severely pathological symptom at admission and did not improve significantly (*p* = 0.197). Diarrhea and reflux were, on average, not pathological at admission and remained stable during treatment. In addition to treatment time, the strongest predictors were ED pathology at admission for the development of abdominal pain, constipation, reflux, and total GI symptoms; stress for the development of constipation and total GI symptoms; and depression for constipation.

**Conclusions:**

Informing patients with AN about the course of GI symptoms and their improvement during weight rehabilitation may help support compliance during treatment.

## Introduction

Eating disorders (EDs) such as anorexia nervosa (AN) are characterized by aberrant patterns of eating behavior. AN is characterized by a restriction in food intake, which can sometimes be combined with binge-purging behavior, high levels of physical activity, and body image disturbances (1–3). Aside from that, gastrointestinal (GI) complaints are not only common in patients with AN (4) but also play an important role in the maintenance of eating disorder pathology (5) as patients often report GI symptoms as a justification for not being able to eat. Patients with AN show a wide range of GI symptoms, such as abdominal pain, constipation, or heartburn (6, 7). These GI symptoms are thought to be of functional or somatic origin or a combination of both (8–11).

Many of the GI symptoms found in patients with AN overlap with symptoms typical for functional gastrointestinal disorders (FGIDs) (12–14), which is why many authors estimate the functional origin of GI problems in EDs more probable than indicating an underlying somatic GI disease (15, 16). Several studies on FGIDs in patients with ED found a prevalence of more than 90% according to Rome criteria (17, 18). Moreover, disordered eating attitudes are high in patients with irritable bowel syndrome (IBS) (19) and correlate with current IBS symptoms (10). However, some somatic GI disorders can be misleadingly held for an ED, such as inflammatory bowel diseases, achalasia, or celiac disease (20–22), while contrariwise, serious somatic complications requiring a surgical approach or with a possibly lethal outcome can develop by the end of an ED, for example, gastric rupture after a binge-eating episode (6). The microbiota–gut–brain axis is presumed to be an important underlying mechanism for the intensification of GI disturbances such as intestinal microbiota, which have been proven to be altered in patients with AN (23, 24) as well as with FGIDs (25), and play a key role in the bidirectional communication between gut and brain (26, 27).

Overall, many studies report a change in GI disturbances during treatment—some remain the same, some improve or even disappear, and others newly occur—which appears to be particularly dependent on a specific GI symptom (6). Examining studies that analyzed the development of FGIDs, especially AN, evidence can be found that FGIDs persist even after recovery from an ED (28). Nevertheless, other studies show that improvement in overall GI disturbances is possible (29). The key to GI improvement in AN appears to be long-term rehabilitation concerning both weight and psychological condition as symptoms tend to relapse after a short-term weight gain (30).

Predictors of the prevalence of GI problems and development during treatment in patients with AN were analyzed by several studies. Psychopathological features in patients with AN were found to be associated with both functional and somatic GI disorders (31, 32). Starvation, somatization, state–trait anxiety, binge-eating behavior, and laxative abuse were identified as more specific predictors for prevalence of individual FGID subgroups in patients with AN and other EDs (18, 33, 34). In general, frequently examined predictors of worsening of FGIDs or FGID-like symptoms in patients with AN are overall disordered psychological features and diagnosed mental comorbidities like affective (e.g., depression), anxiety, or personality disorders (e.g., obsessive-compulsive, avoidant, and schizoid personalities) (33, 35). Even after recovery from an ED, FGID symptoms more probably persist in patients who are psychologically distressed (28), which underlines the importance of psychotherapy in ED treatment. Moreover, only long-term weight rehabilitation was found to improve both psychological features and GI problems (30). Normal scores of somatization, neuroticism, and anxiety (33), as well as hypochondriasis and depression (36), could be identified as specific psychopathological traits that predict long-term improvement of GI symptoms. Thus far, this has only been shown in patients with bulimia nervosa, but not in patients with AN. Chami et al. reported that GI symptoms only improved in patients with AN who also showed an improvement in depressive symptoms during treatment (37). Depression as predictor of GI development in patients with AN was, to our knowledge, only reported by Salvioli et al. who analyzed GI disturbances at 1- and 6-month follow-ups (36). Nevertheless, Boyd et al. could not identify depression as a significant predictor of the presence of FGID in patients with AN and other EDs (33). Another possible predictive factor of GI symptoms in patients with AN is serum amylase. Several previous studies have found increased serum amylase levels in patients with AN, particularly in binge-purging-type AN (38). However, the relationship between serum amylase and GI symptoms, particularly symptom complexes including reflux and heartburn, which are frequent in patients with binge-purging behavior, has not yet been examined.

Giving patients a deeper understanding of the development of their GI symptoms in the timecourse of nutritional rehabilitation may support the recovery process by helping to motivate and reassure patients with AN to continue to eat normal amounts of food, despite having severe GI complaints at the beginning of treatment. A more closely monitored longitudinal observation of GI symptoms, which is lacking in the current literature, might contribute to this understanding. The aims of the study were to prospectively analyze (i) the timecourse of various GI symptoms in inpatients with AN and (ii) the relationship of the development of GI symptoms during inpatient treatment with anthropometric, demographic, disease-specific, and psychological factors.

## Materials and methods

### Study design and participants

Participants with AN were recruited within the context of an inpatient treatment program at the Eating Disorders Unit of the Department of Psychosomatic Medicine and Psychotherapy at the University Medical Hospital in Tübingen, Germany, in the periods from 2016/01 to 2017/01 and 2018/01 to 2019/02. The main inclusion criterion was a diagnosis of AN according to the Diagnostic and Statistical Manual of Mental Disorders 5 (DSM-5) (1) and the International Classification of Diseases 11 (ICD-11) criteria (2). At admission, all patients were assessed for possible somatic GI diseases that can mimic an ED by using clinical evaluation, blood tests, and GI-specific instrumental investigations, if deemed necessary by the psychologists and physicians in charge. The treatment was conducted by a multidisciplinary team and aimed at increasing body weight and normalizing eating behavior in accordance with the latest developments in medicine as documented in the national German S3-guideline for the assessment and therapy of EDs. This includes a nutritional rehabilitation program and a multi-modal psychotherapeutic approach. At admission, the patients were given normal nutritionally balanced food. Meals were eaten in groups under supervision and with the support from specialized nurses and therapists. The caloric intake was then increased step by step to hypercaloric ranges in order to ensure weight gain. The patients agreed to sign a “weight contract” declaring that they aim to gain a certain amount of weight per week, usually between 500 and 700 grams. Nutritional rehabilitation was further supported by different forms of psychotherapy, for example, individual and group therapy, also complemented by music and art therapy. Time of inpatient treatment usually was planned for 8–10 weeks but sometimes was extended, if considered necessary.

Within the 1st week after admission, patients with AN with a body mass index (BMI) ≤ 18.5 kg/m^2^ were recruited for the study. BMI was calculated using the common formula: BMI = weight/height^2^ (in kg/m^2^). The participants had to be old enough to be treated in the ED unit for adults, which usually requires the age of 18 years. However, patients who would soon be turning 18 years were also admitted in some cases and were not excluded from analysis for reasons of age. Both sexes were included in order to capture the spectrum of patients with AN. Patients with severe somatic diseases, which are likely to induce GI symptoms like Crohn’s disease, or those with limited knowledge of the German language were excluded from analysis.

### Procedures

At admission, body height, age, sex, age at the first diagnosis, duration of illness, vomiting behavior, and laxative misuse were assessed. A total of four questionnaires were distributed at admission and at discharge, assessing patients’ mental condition (psychometric variables): the Eating Disorder Inventory 2 (EDI-2), the Generalized Anxiety Disorder 7 (GAD-7), the 9-item depression scale of the Patient Health Questionnaire (PHQ-9), and the Perceived Stress Questionnaire (PSQ). Only the sum scores of these questionnaires were applied, but no subscales. Body weight and the Gastrointestinal Symptom Rating Scale (GSRS) questionnaire were assessed once weekly from the 1st week after admission until discharge. In case of the GSRS, the subscales were also of interest on top of the total score for better differentiation and analysis of GI symptoms. Serum amylase levels were retrieved from the standard laboratory blood measurements. Finally, the length of inpatient stay was recorded.

*The Gastrointestinal Symptom Rating Scale (GSRS)* is a self-report questionnaire evaluating common GI symptoms (39). The GSRS questionnaire, which has been used in many studies examining patients with FGIDs, is valid and reliable (40) but also brief and therefore useful as an instrument for repeated measures (28). It inquires after patients’ complaints with 15 different items grouped into five subscales on a 7-point Likert scale with scores from 1 denoting “no discomfort at all” to 7 indicating “very severe discomfort.” The conventional total GSRS score is the mean of these 15 symptoms. The five subscales, each constructed of two to four different GI symptoms which are assessed in the questionnaire (with individual symptoms in brackets also serving as a definition for the symptom complexes), are abdominal pain (abdominal pain, hunger pains, nausea), constipation (constipation, hard stools, feeling of incomplete evacuation), diarrhea (diarrhea, loose stools, urgent need for defecation), indigestion (borborygmus, abdominal distension, eructation, flatulence), and reflux (heartburn, acid regurgitation). According to previous studies, a value of ≥ 2 in GSRS scores is defined as pathological (40–42). An AN-typical total score was calculated in addition to the conventional GSRS total score and subscales. This score consisted of the mean of the three GSRS subscales, which had been pathological in the majority of patients at the beginning of treatment (abdominal pain, constipation, and indigestion) and were therefore the most relevant GI symptoms in the examined patients with AN.

*The Eating Disorder Inventory 2 (EDI-2)* examines behaviors and attitudes typical for EDs (43). It consists of 91 items divided into the following 11 subscales: asceticism, body dissatisfaction, bulimia, drive for thinness, impulse regulation, ineffectiveness, interoceptive awareness, interpersonal distrust, maturity fears, perfectionism, and social insecurity and has been applied in patients with AN before (44). The six-point response format ranges from 1 denoting “never” to 6 denoting “always.” Answers from all items are summed up to a total score. Only the EDI-2 total scores were used for further analysis as ED pathology in general was of interest.

*The Generalized Anxiety Disorder 7 (GAD-7)* is a seven-item anxiety scale of the Patient Health Questionnaire, which was developed and initially validated as a brief self-report screening tool for generalized anxiety disorder (GAD) in clinical practice (45). Answers are scored from 0 to 3, indicating increasing symptoms with increasing values, and are summed up to a total score. Apart from that, it is also commonly applied as a reliable and valid tool in order to measure general anxiety symptoms in different populations and settings (46).

*The Patient Health Questionnaire 9 (PHQ-9)* is a 9-item depression scale based on the Patient Health Questionnaire (47). Answers are scored from 0 to 3, with the latter indicating difficulties nearly on a daily basis. The questions inquire about the patients’ interest or joy in doing things, feeling down or hopeless, difficulty with sleeping, tiredness or feeling of low energy levels, change in appetite, self-perception, ability to concentrate, de-/accelerated functioning, and suicidal thoughts (48). Answers from all items are summed up to a total score.

*The Perceived Stress Questionnaire (PSQ)* is a 30-item stress questionnaire constructed to measure subjective perception, evaluation, and processing of stress (49). The original version of the PSQ contains 30 items. To increase the feasibility of completing the questionnaires, we applied a 20-item short version that evaluates the four subscales worries, tension, demands, and joy. It applies five items each on a four-item Likert scale, with 1 denoting “almost never” and 4 denoting “most of the time” within the last 6 weeks (49). The polarity of the items (e.g., feeling calm versus feeling frustrated) was taken into account by respective poling. PSQ values range between 0 and 1 according to common calculation. The PSQ is widely considered to be a reliable and valid tool in the assessment of subjectively experienced stress (50).

### Data analysis

Data were analyzed using IBM SPSS Statistics, version 27.0 (51) and R statistics (52). Data sets of participants were included if GSRS questionnaires were completed at least three times of the seven measurements during the first 6 weeks of their inpatient stay so that a development would be detectable. For all variables, mean, standard deviation (SD), median, and interquartile range (IQR) at the beginning of treatment were calculated. The percentage of patients with pathological GSRS scores at admission was also recorded.

Because of the repeated measures design of the study with measures at different timepoints (level 1) nested within patients (level 2), a mixed linear model (MLM) approach, also called hierarchical linear model, was considered appropriate to analyze the data. The MLM takes into account that the repeated observations in one patient are dependent. If calculated otherwise, estimators could be biased, and standard errors (SE) could be underestimated. In case of missing data, imputation is not necessary as cases are not excluded listwise, and parameters are estimated with the available data. Within- and between-person predictors were handled following common recommendations (53). Because of meeting the linearity of relationships, data transformation was not required.

MLM can be used with different covariance types depending on the structure of the data. The data showed different variance at each timepoint and constant covariance between the measurements; thus, a diagonal covariance structure was applied. Several different models were built in order to analyze the data step by step. (1) A so-called “empty model” without any explanatory variables was calculated to estimate the extent to which the GSRS scores differed between patients. (2) Next, the random intercept model was conducted with the number of treatment weeks, which is the central explanatory predictor at level 1, as the fixed effect. (3) A random slope model was calculated to assess for varying time slopes across patients. The latter two steps were also tested with a quadratic slope by squaring the amount of treatment weeks to test for increasing or decreasing influence of treatment time on the GI development. (4) BMI and amylase, the remaining predictors of level 1 with measures at each timepoint, were applied as fixed effects in separate models. (5) All predictors of level 2 (those which were only measured at the beginning of treatment) were modeled as fixed effects in separate models. Interactions of all predictors with the treatment time in weeks were tested consecutively to examine if the influence of a predictor on GI symptom development changes during treatment time.

Model fits calculated as -2 log-likelihood and Akaike information criterion were taken into account in order to compare the fit of different models. MLM was used for the total GSRS score and each subscale separately using repeated measures until week 10. Week 10 was chosen as the last timepoint to be included into analysis as this was the upper limit of the planned treatment time and also matched with the approximate average time of the actual inpatient stay.

MLM calculations were not adjusted for multiple testing, but only strong *p* values of < 0.005 were considered as statistically significant. Results with *p* values < 0.05 were, however, also reported and can be considered putative effects.

## Results

### Characteristics of participants

Of the 38 patients with diagnosis of AN included in the study, 17 patients were categorized as restrictive, 16 as binge-purging, and five as unspecified subtype. The average age was 27.9 [SD = 10.3, median = 23.5, IQR = (20.0–35.0)] years, ranging from 17 to 53 years. Only two male patients fulfilled the inclusion criteria (5.3% of patients); 19 patients were categorized as having extreme AN with a BMI of < 15.0 kg/m^2^ according to the DSM-5 criteria, nine as severe (BMI 15.0–15.9 kg/m^2^), three as moderate (BMI 16.0–16.9 kg/m^2^), and six as mild AN (BMI > 17.0 kg/m^2^) (1). The average length of inpatient stay was 9.5 [SD = 3.3, median = 9.0, IQR = (7.0–12.0)] weeks, ranging from 5 to 17 weeks. On average, the mean age at first diagnosis was 20.9 [SD = 9.1, median = 18.0, IQR = (15.0–22.0)] years, and duration of the disease was 7.5 [SD = 8.5, median = 4.0, IQR = (2.5–10.0)] years. Vomiting behavior was reported by 11 (29.0%) and laxative misuse by seven patients (18.4%). Most patients had several somatic and mental comorbidities; depressive disorder was the most frequent mental disorder, found in 32 patients. Other comorbidities were posttraumatic stress disorder in four patients and obsessive-compulsive disorders in three patients; one patient had attempted suicide twice, and another had surgically treated ulcerative colitis.

### Gastrointestinal development over the timecourse

The pre- and post-treatment characteristics of the study population are summarized in Table 1. A more detailed descriptive overview on the longitudinal GI data is given in Supplementary Table 1. In the median, 31.0 [IQR = (20.0–34.5)] measurements were available per timepoint. The prevalence of GI symptoms at admission varied widely with diarrhea being quite rare (26.3%), while relevant symptoms in the field of indigestion were reported by almost every patient (94.7%). Abdominal pain and constipation were similarly highly prevalent (76.3% and 84.2%, respectively), whereas reflux was a serious problem in approximately half of the participants (42.1%). Overall GI symptoms scored pathological in 86.8% of the patients. As described earlier, we constructed an additional AN-typical GSRS total score summarizing the means of abdominal pain, constipation, and indigestion as these were, on average, pathological at the beginning of treatment and thus the most relevant GI symptoms in the examined AN population, being pathological in 86.8% of patients at the beginning of treatment.

**TABLE 1:** Description of body mass index, serum amylase levels, gastrointestinal symptom rating scale scores, and psychometric variables at admission and discharge.

	Admission			Discharge			Difference	
			
	Mean (SD)	Median [IQR]	[Min-Max]	Mean (SD)	Median (IQR)	[Min-Max]	Mean	Delta Mean in %
BMI	14.73 (1.79)	14.92 [13.22–14.92]	[11.42–18.14]	16.55 (1.81)	17.06 (2.67)	[13.02–19.84]	1.81	12.27
Amylase	108.21 (68.16)	92.00 [71.00–113.25]	[32.00–330.00)	108.73 (51.02)	89.00 (54.50)	[53.00–264.00]	0.53	0.49
GSRS								
Total	3.06 (1.12)	2.73 [2.14–4.10]	[1.60–5.60]	2.58 (1.14)	2.07 (1.77)	[1.20–4.80]	0.49	15.85
AN-typical	3.49 (1.29)	2.99 [2.56–4.33]	[1.64–6.42]	2.97 (1.37)	2.57 [1.83–3,95]	[1.25–6.55]	0.52	14.89
Abdominal Pain	3.07 (1.57)	2.67 [1.67–4.17]	[1.00–6.67]	2.40 (1.12)	2.00 (2.17)	[1.00–4.67]	0.66	21.58
Constipation	3.61 (1.88)	3.00 [2.17–5.17]	[1.00–7.00]	3.01 (1.82)	2.67 (3.25)	[1.00–7.00]	0.60	16.64
Diarrhea	2.09 (1.60)	1.33 [1.00–3.00]	[1.00–7.00]	1.89 (1.27)	1.33 (1.58)	[1.00–6.00]	0.20	9.60
Indigestion	3.93 (1.34)	3.75 [2.75–4.75]	[2.00–7.00]	3.38 (1.52)	3.00 (2.38)	[1.50–6.75]	0.55	13.89
Reflux	1.94 (1.29)	1.00 [1.00–3.00]	[1.00–6.00]	1.59 (0.94)	1.00 (1.00)	[1.00–4.50]	0.35	18.08
Psychometric variables								
EDI-2	288.59 (70.20)	276.50 [230.26–348.75]	[173.00–416.00]	282.28 (70.93)	296.00 (131.50)	[144.00–389.00]	6.31	2.19
GAD-7	15.29 (6.51)	14.00 [9.75–20.00]	[4.00–27.00]	10.97 (6.33)	10.00 (10.00)	[1.00–25.00]	4.33	28.30
PHQ-9	10.76 (5.29)	9.50 [6.00–15.25]	[3.00–21.00]	8.21 (5.27)	6.00 (10.00)	[2.00–17.00]	2.56	23.76
PSQ	0.63 (0.20)	0.68 [0.47–0.77]	[0.23–0.93]	0.51 (0.20)	0.50 (0.28)	[0.03–0.82]	0.11	17.84

AN, Anorexia nervosa; BMI, Body Mass Index (BMI); EDI-2, Eating-Disorder-Inventory-2; GAD-7, Generalized-Anxiety-Disorder-7; GSRS, Gastrointestinal Symptom Rating Scale; IQR, interquartile range; Max, Maximum; Min, Minimum; PHQ-9, Patient-Health-Questionnaire-9; PSQ, Perceived Stress Questionnaire; SD, standard deviation.

**FIGURE 1:**
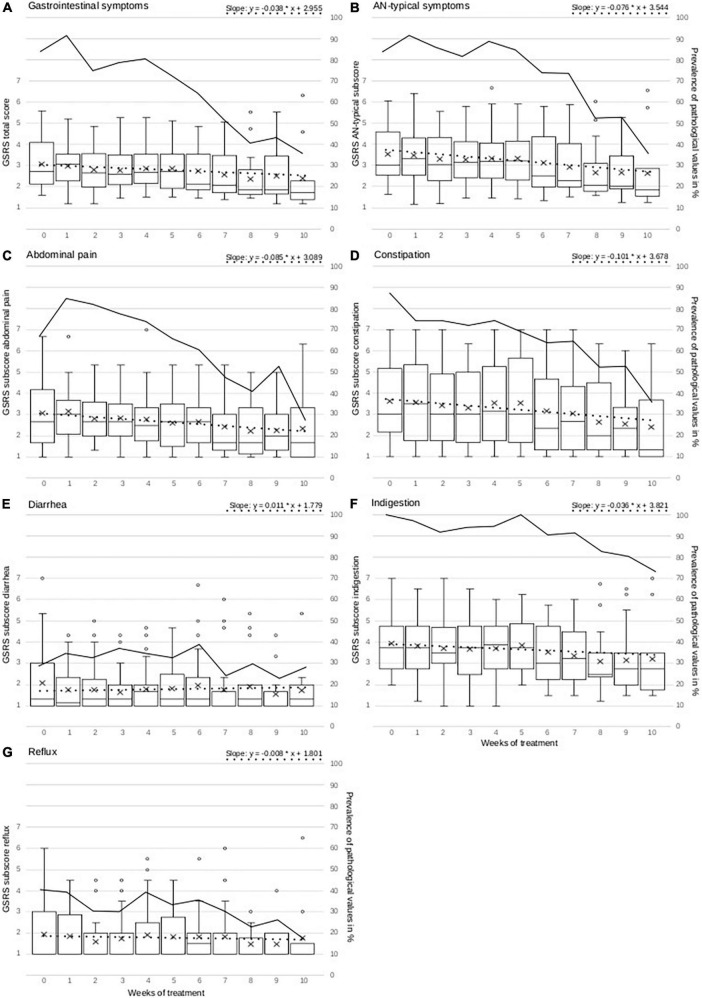
Figure presents weekly measurements of overall GI symptoms, AN-typical GSRS score and GSRS subscales abdominal pain, constipation, diarrhea, indigestion, and reflux as boxplots with mean (+), median (–), interquartile range (IQR = box), whiskers (1.5 × IQR), and outliers (o > 1.5 IQR). The y-axis for all GSRS scores is located on the left of each graph. The dotted line represents the development estimated by mixed linear models for each GSRS score. The mathematical function for this development estimated by mixed linear models is given in the upper right of each separate graph and is constructed with the estimate at admission and the weekly GI change (also see Table 2). In this formula, the *x* value can be replaced by the treatment week in order to calculate the corresponding estimated GSRS score, which is represented by the *y* value, for this specific treatment week. The formula is derived from the mixed linear model results, which are presented in Table 2. The continuous line represents the development of the prevalence of pathological GSRS values in % within the examined population. The y-axis of the prevalence is located on the right side of each graph.

As the GI development over the timecourse was modeled using the MLM, random intercepts (which are estimates at admission), linear and quadratic fixed and random slopes could be tested in addition to the analysis of weekly development. First of all, models with random intercepts and fixed slopes had good model fits, and random intercepts were significant with *p* < 0.001 in all models calculating random intercepts, irrespective of the GSRS score of the dependent variable, as presented in Table 2. The estimates of the random intercepts in the final models range from 1.6 (*p* < 0.001, SE = 0.132) on the GSRS score for diarrhea (not pathological) to a highly pathological value of 3.8 (*p* < 0.001, SE = 0.205) for indigestion. These estimates are also similar to the means at admission, which are presented in Figure 1. Testing of a quadratic development of GI symptoms was not found to be significant for any GSRS score. Temporal development can therefore not be thought to take a quadratic course but is most likely linear among all patients and GSRS scores. In the next step, models allowing for GI development differing between the patients over the timecourse in addition to random intercepts (random slope models) were tested. This further improved the model fits of the models examining the GSRS total and AN-typical scores as well as the subscales abdominal pain, constipation, and indigestion, but not diarrhea and reflux, which are therefore the only subscales reported as models with fixed slopes in Table 2. Indigestion and overall GI symptoms (including diarrhea and reflux, which were overall not pathological in the first place) did not change significantly, as can be seen in Table 2. However, abdominal pain, constipation, and AN-typical GI symptoms improved significantly during treatment with estimated weekly improvements of 0.085, 0.101, and 0.076 on the GSRS score. The estimated improvements after 10 weeks of treatment are shown in Figure 1, for example, in the case of constipation, one point on the GSRS score is estimated to be improved after 10 weeks of treatment. Reaching normal values is still estimated by the models to take several weeks longer depending on the symptom. As presented in Figure 1, at the end of the treatment, more patients range within GSRS scores considered healthy than at the beginning for all total and subscales, except diarrhea. Applying the functions given in Figure 1, the week in which GSRS scores are estimated to reach normal values can also be estimated. For the GSRS scores which improve significantly, namely, AN-typical, abdominal pain, and constipation, 20.3, 12.8, and 16.6 treatment weeks are estimated to be necessary in order to reach normal values for the average patient population. However, this is no longer included in our observation period and should therefore be considered with caution. Diarrhea and reflux remained stable over the timecourse, with the median of values being already below pathological threshold at admission. Still, especially in reflux, many outliers showing much higher values than both the median and the mean account for a more diverse picture.

**TABLE 2:** Key findings of mixed linear models for gastrointestinal symptoms rating scale total and subscores.

	Total GSRS		AN-typical		Abdominal pain		Constipation		Diarrhea		Indigestion		Reflux	
							
	Estimate (SE) CI	*p* value	Estimate (SE) CI	*p* value	Estimate (SE) CI	*p* value	Estimate (SE) CI	*p* value	Estimate (SE) CI	*p* value	Estimate (SE) CI	*p* value	Estimate (SE) CI	*p* value
Estimate at admission	2.955*** (0.153) [2.644–3.265]	< 0.001	3.544*** (0.193) [3.153–3.936]	< 0.001	3.089*** (0.174) [2.740–3.440]	< 0.001	3.678*** (0.293) [3.086–4.271]	< 0.001	1.779*** (0.159) [1.460–2.098]	< 0.001	3.821*** (0.205) [3.406–4.237]	< 0.001	1.801*** (0.158) [1.484–2.119]	< 0.001
Weekly GI change	−0.038 (0.021) [−0.081–0.005]	0.079	−0.076** (0.025) [−0.127–−0.024]	0.005	−0.085** (0.024) [−0.134–−0.035]	0.002	−0.101** (0.032) [−0.167–−0.035]	0.004	0.011 (0.015) [−0.020–0.042]	0.479	−0.036 (0.028) [−0.093–0.020]	0.197	−0.008 (0.014) [−0.036–0.019]	0.561
Predictor														
Weekly BMI	−0.011 (0.065) [−0.141–0.118]	0.863	0.012 (0.081) [−0.150–0.174]	0.882	0.034 (0.083) [−0.131–0.198]	0.684	0.088 (0.126) [−0.163–0.338]	0.489	0.009 (0.077) [−0.144–0.163]	0.904	−0.000 (0.089) [−0.178–0.177]	0.996	0.134 (0.076) [−0.018–0.286]	0.083
Weekly Amylase	0.003 (0.002) [−0.001–0.008]	0.159	0.006* (0.003) [0.000–0.011]	0.043	0.002 (0.003) [−0.005–0.008]	0.557	0.008 (0.004) [−0.001–0.017]	0.066	0.002 (0.003) [−0.004–0.008]	0.493	0.000 (0.003) [−0.006–0.006]	0.975	−0.001 (0.003) [−0.006–0.005]	0.843
Age	−0.012 (0.015) [−0.043–0.019]	0.427	−0.012 (0.019) [−0.051–0.027]	0.540	−0.021 (0.017) [−0.055–0.014]	0.232	0.010 (0.029) [−0.049–0.070]	0.725	−0.002 (0.016) [−0.034–0.030]	0.908	−0.019 (0.021) [−0.060–0.023]	0.373	−0.026 (0.016) [−0.058–0.006]	0.107
Duration of illness	0.002 (0.020) [−0.039–0.044]	0.907	0.014 (0.026) [−0.038–0.067]	0.576	−0.004 (0.024) [−0.053–0.046]	0.877	0.050 (0.037) [−0.025–0.125]	0.185	−0.021 (0.023) [−0.066–0.025]	0.371	−0.001 (0.028) [−0.056–0.059]	0.958	−0.022 (0.022) [−0.067–0.0236]	0.341
Age at first diagnosis	−0.007 (0.019) [−0.045–0.031]	0.695	−0.011 (0.023) [−0.059–0.036]	0.631	−0.010 (0.022) [−0.054–0.035]	0.662	−0.011 (0.034) [−0.080–0.058]	0.750	0.009 (0.020) [−0.030–0.049]	0.634	−0.001 (0.026) [−0.053–0.051]	0.955	−0.025 (0.020) [−0.065–0.015]	0.216
Length of inpatient stay	0.006 (0.049) [−0.093–0.105]	0.905	0.013 (0.062) [−0.112–0.137]	0.840	−0.043 (0.055) [−0.154–0.068]	0.435	0.042 (0.093) [−0.146–0.231]	0.653	−0.006 (0.050) [−0.107–0.095]	0.901	0.038 (0.065) [−0.095–0.170]	0.566	−0.057 (0.050) [−0.159–0.044]	0.262
Laxative misuse	−0.406 (0.349) [−1.120–0.307]	0.254	−0.399 (0.451) [−1.321–0.523]	0.384	−0.312 (0.421) [−1.166–0.542]	0.463	0.165 (0.735) [−1.335–1.665]	0.824	−0.190 (0.308) [−0.812–0.432]	0.541	−1.022 (0.534) [−2.114–0.069]	0.065	0.029 (0.368) [−0.717–0.775]	0.937
Vomiting behavior	0.376 (0.333) [−0.300–1.051]	0.267	0.297 (0.424) [−0.563–1.158]	0.488	0.360 (0.385) [−0.416–1.136]	0.354	0.330 (0.647) [−0.980–1.640]	0.613	0.588 (0.330) [−0.074–1.250]	0.081	0.239 (0.454) [−0.681–1.159]	0.602	0.082 (0.355) [−0.631–0.795]	0.818
Eating behavior (EDI-2)	0.007** (0.002) [0.003–0.012]	0.001	0.010*** (0.003) [0.005–0.015]	< 0.001	0.009** (0.003) [−0.003–0.014]	0.002	0.016*** (0.004) [0.008–0.023]	< 0.001	0.002 (0.003) [−0.003–0.007]	0.519	0.005 (0.003) [−0.001–0.011]	0.121	0.007** (0.002) [0.002–0.011]	0.004
Depression (PHQ-9)	0.059* (0.024) [0.010–0.109]	0.020	0.086* (0.030) [0.025–0.147]	0.007	0.080* (0.029) [0.022–0.137]	0.008	0.156*** (0.041) [0.071–0.241]	< 0.001	0.003 (0.028) [−0.052–0.059]	–0.906	0.021 (0.035) [−0.050–0.093]	0.551	0.024 (0.026) [−0.029–0.077]	0.368
Anxiety (GAD-7)	0.048 (0.031) [−0.015–0.111]	0.133	0.059 (0.039) [−0.021–0.139]	0.144	0.055 (0.037) [−0.019–0.130]	0.139	0.145* (0.055) [0.033–0.257]	0.013	0.025 (0.033) [−0.042–0.093]	0.450	−0.013 (0.043) [−0.101–0.075]	0.760	0.048 (0.032) [−0.016–0.112]	0.137
Stress (PSQ)	2.048* (0.775) [0.470–3.626]	0.013	3.103** (0.933) [1.202–5.004]	0.002	2.200* (0.931) [0.309–4.090]	0.024	5.825*** (1.252) [3.276–8.374]	< 0.001	−0.145 (0.894) [−1.952–1.661]	0.872	1.684 (1.105) [−0.567–3.935]	0.137	0.494 (0.838) [−1.194–2.182]	0.558

The main results of the basic mixed linear models (MLM) analyzing all Gastrointestinal Symptom Rating Scale (GSRS) scales as a dependent variable are presented in the upper part. The estimates at admission and the estimated weekly gastrointestinal (GI) change for all GI symptoms as calculated in the best fitting models are reported. As best fitting models MLM with random slopes were identified for total GSRS score, AN-typical GSRS score and the subscales abdominal pain, constipation and indigestion. MLM with fixed slopes produced the best model fits for the GSRS subscales diarrhea and indigestion. Furthermore, variables tested as predictors for development of GI symptoms calculated with the same MLM as explained above are presented in this table. Standard errors (SE), confidence intervals (CI) and p values are reported for all calculated estimates. Significant p values were indicated with stars: *p < 0.05; **p < 0.005; ***p < 0.001. Due to multiple testing, we only considered p values with p < 0.005 as statistically significant. The predictor’s estimates specify how many points the examined GSRS score changes if the respective predictor changes a full point on its scale. Therefore, taking into account the mean values of the predictors as reported in Table 1 and in the main text is recommended. Interactions between predictor and treatment week were also calculated but not reported here as no interaction was statistically significant with p < 0.005.

### Predictors of gastrointestinal symptom improvement

An overview on the assessed predictors of GI symptom improvement is presented in Table 2. The predictors were analyzed applying the same models as explained before (MLM with random intercepts and fixed slopes for diarrhea and reflux and MLM with random intercepts and random slopes for abdominal pain, constipation, indigestion, and overall AN-typical GI symptoms). Strong predictors with *p* < 0.005 were ED pathology for abdominal pain, constipation, reflux, overall AN-typical GI symptoms, stress for constipation, and AN-typical GI symptoms, as well as depression for constipation. The estimates reported are not effect sizes and not standardized and can therefore not be compared between the different predictors. Instead, they are interpreted in relation with the respective GSRS scores and the average values of the respective predictor. For example, for constipation and ED pathology, this implies that the GSRS score is estimated to be 0.016 GSRS points lower for every full point less on the EDI-2. Given that the average EDI-2 score of our study population is 288.6, substantial differences between the patients in constipation outcomes associated with higher or lower EDI-2 scores appear reasonable.

More factors with *p* < 0.05 but > 0.005 are reported in Table 2 but not discussed further as they cannot safely be considered significant predictors due to multiple testing. Interactions between tested predictors and treatment week, which are supposed to indicate if an effect of a predictor changes during treatment, are not reported in Table 2 because no interaction was found to be significant with *p* < 0.005. Other tested predictors (BMI, serum amylase, age, age at first diagnosis, duration of disease, length of inpatient stay, vomiting behavior, laxative misuse, anxiety, and depression) did not significantly contribute to weekly GI development. Altogether, the most important contributing predictor was treatment time, as reported in the previous section.

## Discussion

GI symptoms are common in patients with AN (6). In line with other studies, we found that such symptoms improved in patients with AN during or after inpatient treatment (11, 54, 55). However, this study analyzed the dynamics of GI symptom development in patients with AN during treatment for the first time—knowledge that can be helpful in supporting patients with AN during nutritional rehabilitation. In addition, this study extends the knowledge about predictors for GI outcome in patients with AN.

Abdominal pain, constipation, and indigestion (borborygmus, abdominal distension, eructation, flatulence) are typical GI symptoms in patients with AN and develop differently in the timecourse of treatment. Abdominal pain and constipation, as well as AN-typical symptoms, altogether improved significantly within the observation period. Constipation appeared to improve at a higher rate than abdominal pain, with about 0.1 points on the GSRS score per treatment week. Both symptom complexes did, on average, not normalize during treatment, but due to significant weekly improvement, the normalization of the average GSRS scores of abdominal pain and constipation can be assumed. However, a longer observation period would be required for certainty. These results can be helpful in encouraging patients to continue eating despite GI symptoms troubling the patients particularly at the beginning of treatment.

Symptoms of indigestion were very high at the beginning of treatment and neither normalized throughout treatment in more than 80% of the patients nor improved significantly. The persistence of these symptoms is consistent with a previous study (54). Apparently, indigestion is the most persistent symptom complex concerning GI discomfort in patients with AN. The question is when, and if at all, indigestion symptoms would normalize and whether this would require a longer observation period since symptoms might persist for years after recovery, as shown in a retrospective study (28). Generally, many studies drew the conclusion that long-term disordered eating behavior can lead to prolonged medical problems and physiological adaptations that may appear as FGIDs (10, 11, 34).

Diarrhea and reflux symptoms played a minor role in the investigated population with AN as the majority of patients showed normal values from admission onward. In the first week of treatment, diarrhea symptoms appeared to worsen in some patients, but not significantly and not to pathological levels on the GSRS. Reflux symptoms were of particular interest as they might be a consequence of binge-purging behavior, more specifically vomiting. Interestingly, neither serum amylase nor vomiting behavior, factors that can be hypothesized to be connected with reflux symptoms, was found to be associated with the latter for the complete AN group. An analysis within the different AN subgroups—restrictive and binge-purging—would have been necessary but would have resulted in insufficient subgroup sample sizes in this study.

A key finding of this study was that disordered ED pathology at admission appeared to be an important predictor for the development of abdominal pain, constipation, reflux, and overall AN-typical GI symptoms. Overall, this suggested that GI symptoms might rather be of functional origin in patients with AN. Although ED pathology appeared to be a predictor for several GI symptoms in this study, the EDI-2 score remained high despite treatment. In line with the high mortality rate of patients with AN due to the difficult course the disease can take, ED pathology was found to be persistent, on average, even after inpatient treatment (56). Surprisingly, weekly measured BMI did not predict the development of GI symptoms. As weight gain is a main objective in the treatment of patients with AN and treatment of AN has been shown to improve GI symptoms, it was expected that a BMI increase would contribute to a better GI outcome and vice versa, although information about BMI as a predictor was not found in the literature beforehand. However, this finding overall implied that GI symptoms might improve equally in patients with different starting positions or suboptimal weight development. Overall, nutritional rehabilitation with normalized food intake and functioning of the GI tract appeared to support a favorable GI outcome with a higher likelihood than mere weight gain in this study. Despite these findings, it might also be possible that the length of observation and thus the degree of weight restoration was not sufficient to find a positive influence of weight gain on GI symptoms as many patients in this study remained on low BMI levels until discharge. However, applying EDI-2 at admission might be useful in order to estimate GI development before treatment and particularly support patients with more severely disordered ED pathology.

In addition, the outcome of constipation was also predicted by stress and depression, and AN-typical GI symptoms could be predicted by stress. Anxiety was the only psychological factor that showed no association with any GI symptom complex—a finding which is coherent with an earlier study (36). Considering depression as a predictor of GI symptom development in AN, we found studies with conflicting results, as presented in Introduction (33, 36). An explanation for this discrepancy of depression as a predictor might be the observation period. It is possible that depression can be considered as a predictor of long-term GI symptom improvement, whereas depression may not be a considerable predictor when GI symptoms are examined for a short term. Our finding is also consistent with a that of a previous study that reported GI symptoms to improve in patients with AN only if depressive symptoms improved coherently (37). This supports the hypothesis expressed by several authors that GI symptoms and EDs like AN might be mediated by an underlying psychiatric disorder and therefore regularly occur together (28), while others also interpret the coherent appearance of disordered ED pathology, GI disturbances, and psychiatric disorders as an overlapping psychopathophysiologic syndrome (57). Specifically in cases of severe constipation, the assessment of the prevalence of stress and depression could be helpful for the treatment of an affected patient.

This study has strengths and limitations. First, it is possible that patients with ED report more severe GI symptoms than they actually have; hence, using this as a means to justify why eating is not possible. Thus, the reliance on self-report data may bias the results. Of course, complaints about GI symptoms should be taken seriously in patients with AN, but there still might be a certain degree of overestimation additionally because of psychomental distress like depression, which is prevalent in many patients with AN. Second, replicating the analyses of this study with a larger sample size would be necessary in order to strengthen the results. This would be helpful for the analyses conducted in general but also because an addition of separate analyses comprising the different subtypes of AN would be feasible. Such subgroup analyses have been left out in this study because this would have resulted in insufficient subgroup sample sizes. However, they could support the understanding of, for instance, the relationship between binge-purging behavior, reflux symptoms, and increased serum amylase levels in future studies. Third, some of the patients included received medications like antidepressants because of depressive symptoms. However, some antidepressants, among them serotonin reuptake inhibitors, can influence gut motility and thus GI symptoms directly and not only *via* improving depression (58). Fourth, this study did not include a healthy control group. Finally, there are also special strengths of this study. One of these is the measurement of GI symptoms in regular periods over 10 weeks and under controlled conditions in inpatients with AN which was conducted as such for the first time. This provided the possibility of a close monitoring of the GI symptom development over the timecourse. Another strength is the MLM approach as a rather complex method of analysis, which enabled analyzing data with repeated measures design and also including predictors.

In summary, abdominal pain, and constipation-related and AN-typical GI symptoms improved significantly and stabilized in most patients to a normal range during the treatment period. Diarrhea- and reflux-related symptoms overall played a less important role in patients with AN with normal values on average throughout treatment and therefore did not improve significantly during the timecourse. Disordered ED pathology at admission predicted the outcome of abdominal pain, constipation, and reflux, as well as AN-typical and overall GI symptoms; depression predicted the outcome of constipation symptoms; and stress predicted the outcome of constipation and AN-typical symptoms. Weekly measured BMI, serum amylase, anxiety, current age, age at first diagnosis, duration of illness, length of inpatient stay, vomiting behavior, or laxative misuse were not found to predict any GI outcomes. Further research comprising larger sample sizes in order to strengthen the results and enable analyses of different AN subgroups would be a necessary contribution to the field.

## Data availability statement

The raw data supporting the conclusions of this article will be made available by the authors, without undue reservation.

## Ethics statement

The studies involving human participants were reviewed and approved by Ethics Committee of the University of Tübingen (ethic protocol number: 392/2019BO2). Written informed consent to participate in this study was provided by the participants or the participants’ legal guardian/next of kin in case of underage participants.

## Author contributions

CR was responsible for data analysis and interpretation, drafted the manuscript, and acquired data. IM was responsible for conception, design, and preparation of the study, data interpretation, and manuscript writing. NM was responsible for data interpretation. NS was involved in data management. SZ and PE were responsible for conception and design of the study and data interpretation. KG and AS were involved in data interpretation. All authors revised the manuscript and approved the final version of the manuscript.
