# Erratum: Gastrointestinal complaints in patients with anorexia nervosa in the timecourse of inpatient treatment

**DOI:** 10.3389/fpsyt.2022.1085733

**Published:** 2022-11-25

**Authors:** Frontiers Production Office

**Affiliations:** Frontiers Media SA, Lausanne, Switzerland

**Keywords:** abdominal pain, anorexia nervosa, constipation, eating disorders, gastrointestinal complaints, indigestion

Due to a production error, an author's comment was added to the article in error.

A correction has been made to the section **Discussion**, Paragraph Number 8:

“In summary, abdominal pain, and constipation-related and AN-typical GI symptoms improved significantly and stabilized in most patients to a normal range during the treatment period. Diarrhea- and reflux-related symptoms overall played a less important role in patients with AN with normal values on average throughout treatment and therefore did not improve significantly during the timecourse. Disordered ED pathology at admission predicted the outcome of abdominal pain, constipation, and reflux, as well as AN-typical and overall GI symptoms; depression predicted the outcome of constipation symptoms; and stress predicted the outcome of constipation and AN-typical symptoms. Weekly measured BMI, serum amylase, anxiety, current age, age at first diagnosis, duration of illness, length of inpatient stay, vomiting behavior, or laxative misuse were not found to predict any GI outcomes. Further research comprising larger sample sizes in order to strengthen the results and enable analyses of different AN subgroups would be a necessary contribution to the field.”

Due to a production error, a word was added which changed the meaning of a sentence.

A correction has been made to the section **Results**, *Predictors of gastrointestinal symptom improvement*, Paragraph Number 2:

“More factors with *p* < 0.05 but > 0.005 are reported in Table 2 but not discussed further as they cannot safely be considered significant predictors due to multiple testing. Interactions between tested predictors and treatment week, which are supposed to indicate if an effect of a predictor changes during treatment, are not reported in Table 2 because no interaction was found to be significant with *p* < 0.005. Other tested predictors (BMI, serum amylase, age, age at first diagnosis, duration of disease, length of inpatient stay, vomiting behavior, laxative misuse, anxiety, and depression) did not significantly contribute to weekly GI development. Altogether, the most important contributing predictor was treatment time, as reported in the previous section.”

The publisher apologizes for these mistakes. The original article has been updated.

