# Supplementary material for: Gastrointestinal complaints in patients with anorexia nervosa in the timecourse of inpatient treatment
Source: Front Psychiatry. 2022 Aug 18;13:962837. doi: 10.3389/fpsyt.2022.962837 (PMC9436028; doi:10.3389/fpsyt.2022.962837)
Supplement: Supplementary file 1 [file Table_1.docx]

| **Supplementary Table 1 – Descriptive statistics of GSRS total score and subscales of all weeks from admission to treatment week 10** | | | | | | | |
| --- | --- | --- | --- | --- | --- | --- | --- |
|  |  |  |  |  |  |  |  |
|  | **Total GSRS** | **AN-typical** | **Abdominal Pain** | **Constipation** | **Diarrhea** | **Indigestion** | **Reflux** |
|  |  |  |  |  |  |  |  |
|  | Mean (SD)  Median [IQR] | Mean (SD)  Median [IQR] | Mean (SD)  Median [IQR] | Mean (SD)  Median [IQR] | Mean (SD)  Median [IQR] | Mean (SD)  Median [IQR] | Mean (SD)  Median [IQR] |
|  |  |  |  |  |  |  |  |
| Admission | 3.061 (1.124)  2.733 [2.133-4.100] | 3.537 (1.333)  3.000 [2.528-4.597] | 3.067 (1.567)  2.667 [1.667-4.167] | 3.613 (1.880)  3.000 [2.167-5.167] | 2.093 (1.603)  1.333 [1.000-3.000] | 3.930 (1.336)  3.750 [2.750-4.750] | 1.940 (1.294)  1.000 [1.000-3.000] |
| Week 1 | 2.992 (0.905)  3.067 [2.267-3.533] | 3.557 (1.247)  3.361 [2.583-4.333] | 3.167 (1.279)  3.000 [2.083-3.667] | 3.565 (1.981)  3.500 [1.750-5.333] | 1.759 (0.997)  1.167 [1.000-2.333] | 3.814 (1.318)  3.750 [2.750-4.750] | 1.861 (1.131)  1.000 [1.000-2.875] |
| Week 2 | 2.798 (0.912)  2.667 [2.000-3.567] | 3.308 (1.182)  3.042 [2.285-4.326] | 2.806 (1.070)  2.667 [2.000-3.583] | 3.417 (1.825)  3.000 [1.750-4.917] | 1.769 (1.001)  1.333 [1.000-2.250] | 3.701 (1.366)  3.500 [3.000-4.688] | 1.597 (0.932)  1.000 [1.000-2.000] |
| Week 3 | 2.770 (0.947)  2.600 [2.100-3.500] | 3.278 (1.116)  3.167 [2.431-4.139] | 2.838 (1.077)  2.667 [2.000-3.500] | 3.313 (1.770)  3.000 [1.667-5.000] | 1.636 (0.855)  1.333 [1.000-2.000] | 3.682 (1.317)  3.750 [2.500-4.750] | 1.727 (1.206)  1.000 [1.000-2.000] |
| Week 4 | 2.861 (0.954)  2.700 [2.150-3.533] | 3.335 (1.183)  3.181 [2.410-4.063] | 2.769 (1.323)  2.500 [1.750-3.333] | 3.528 (1.961)  3.167 [1.750-5.250] | 1.796 (0.957)  1.667 [1.000-2.000] | 3.708 (1.254)  3.875 [2.750-4.750] | 1.903 (1.297)  1.000 [1.000-2.500] |
| Week 5 | 2.865 (0.954)  2.733 [1.933-3.533] | 3.335 (1.254)  3.222 [2.306-4.167] | 2.606 (1.220)  2.667 [1.500-3.500] | 3.535 (2.149)  3.000 [1.667-5.667] | 1.808 (1.067)  1.333 [1.000-2.500] | 3.864 (1.204)  3.750 [2.750-4.875] | 1.833 (1.210)  1.000 [1.000-2.750] |
| Week 6 | 2.738 (1.068)  2.133 [1.867-3.533] | 3.114 (1.311)  2.500 [1.972-4.361] | 2.656 (1.252)  2.667 [1.667-3.333] | 3.161 (2.120)  2.333 [1.333-4.667] | 1.968 (1.370)  1.333 [1.000-2.333] | 3.524 (1.397)  3.000 [2.250-4.750] | 1.807 (1.101)  1.500 [1.000-2.000] |
| Week 7 | 2.582 (1.131)  2.067 [1.733-3.467] | 2.937 (1.308)  2.278 [1.972-4.361] | 2.420 (1.386)  1.667 [1.333-3.000] | 3.044 (1.950)  2.667 [1.333-4.333] | 1.768 (1.430)  1.000 [1.000-1.667] | 3.348 (1.270)  3.250 [2.250-4.500] | 1.826 (1.362)  1.000 [1.000-2.000] |
| Week 8 | 2.373 (1.179)  1.867 [1.633-2.800] | 2.650 (1.328)  2.083 [1.778-3.097] | 2.235 (1.316)  1.667 [1.167-3.333] | 2.628 (1.814)  2.000 [1.333-4.500] | 1.902 (1.428)  1.333 [1.000-2.000] | 3.088 (1.422)  2.500 [2.375-3.500] | 1.471 (0.649)  1.000 [1.000-1.750] |
| Week 9 | 2.512 (1.310)  1.867 [1.667-3.450] | 2.656 (1.290)  2.000 [1.889-3.444] | 2.244 (1.205)  2.000 [1.333-3.000] | 2.556 (1.592)  2.333 [1.333-3.333] | 1.556 (0.997)  1.000 [1.000-1.667] | 3.167 (1.622)  2.750 [2.000-3.500] | 1.467 (0.812)  1.000 [1.000-2.000] |
| Week 10 | 2.381 (1.607)  1.733 [1.400-2.267] | 2.644 (1.795)  1.833 [1.556-2.861] | 2.333 (1.795)  1.667 [1.000-3.333] | 2.394 (2.054)  1.333 [1.000-3.667] | 1.727 (1.281)  1.333 [1.000-2.000] | 3.205 (1.819)  2.750 [1.750-3.500] | 1.773 (1.679)  1.000 [1.000-1.500] |
|  |  |  |  |  |  |  |  |
| Legend: This supplementary table presents the most important descriptive data presented graphically in Figure 1 of the main document. All means with standard deviations (SD) and medians with interquartile ranges (IQR) of the Gastrointestinal Symptom Rating Scale (GSRS) score and subscales for all measurement points are summarized. | | | | | | | |
